# Cases of acute mercury poisoning by mercury vapor exposure during the demolition of a fluorescent lamp factory

**DOI:** 10.1186/s40557-017-0184-x

**Published:** 2017-06-20

**Authors:** Sang Yoon Do, Chul Gab Lee, Jae Yoon Kim, Young Hoon Moon, Min Sung Kim, In Ho Bae, Han Soo Song

**Affiliations:** 1 Department of Occupational & Environmental Medicine, School of Medicine, Chosun University Hospital, 365 Pilmun-daero Dong-gu, Gwangju, 61453 South Korea; 2 Department of Dermatology, School of Medicine, Chosun University Hospital, 365 Pilmun-daero Dong-gu, Gwangju, 61453 South Korea

**Keywords:** Mercury vapor, Elemental mercury, Occupational exposure, Swan neck deformity, Chloroacne, Fluorescent lamp factory, Korea

## Abstract

**Background:**

In 2015, workers dismantling a fluorescent lamp factory in Korea were affected by mercury poisoning from exposure to mercury vapor.

**Case presentation:**

Eighteen out of the 21 workers who participated in the demolition project presented with symptoms of poisoning and, of these, 10 had persistent symptoms even at 18 months after the initial exposure to mercury vapor. Early symptoms of 18 workers included a general skin rash, pruritus, myalgia, sleep disturbance, and cough and sputum production. Following alleviation of these initial symptoms, late symptoms, such as easy fatigue, insomnia, bad dreams, and anxiety disorder, began to manifest in 10 out of 18 patients. Seven workers underwent psychiatric care owing to sleep disturbance, anxiety disorder, and depression, and three workers underwent dermatologic treatment for hyperpigmentation, erythematous skin eruption, and chloracne-like skin lesions. Furthermore, three workers developed a coarse jerky movement, two had swan neck deformity of the fingers, and two received care at an anesthesiology clinic for paresthesia, such as burning sensation, cold sensation, and pain. Two workers underwent urologic treatment for dysfunction of the urologic system and impotence. However, symptomatic treatment did not result in satisfactory relief of these symptoms.

**Conclusion:**

Awareness of the perils of mercury and prevention of mercury exposure are critical for preventing health hazards caused by mercury vapor. Chelation therapy should be performed promptly following mercury poisoning to minimize damage.

## Background

According to the United Nations Environment Programme (UNEP), global mercury demand has dropped significantly from 9000 tons in the 1960s to 4000 tons in the 1990s. The declining trend persisted until 2005 and demand stabilized in the 2010s. In contrast, mercury emissions and releases mildly increased from 1930 tons in 2005 to 1960 tons in 2010 [1, 2]. The three major areas of elevated mercury emissions and releases were thermal power generation in Asia, particularly China; expansion of artisanal and small-scale gold mining (ASGM), as a result of the rising value of gold and pauperization of rural communities; and disposal of mercury-containing products. ASGM accounts for approximately 37% of global mercury emissions, and approximately 15 million people, including three million women and children, in over 70 countries worldwide are estimated to be ASGM workers. ASGM is an industry with potential risk of elemental mercury poisoning, and it is the leading cause of occupational mercury poisoning [3]. However, in developed countries, disposal or recycling of mercury and unintentional environmental exposure, rather than occupational exposure, are urgent problems. Meanwhile, the current trend of replacing mercury-containing fluorescent lamps with compact fluorescent lamps (CFLs), which contain small amounts of mercury, or light emitting diodes (LEDs), which do not contain any mercury, has reduced the risk of occupational metal mercury poisoning [4]. In Korea, the death of a 15-year-old student owing to mercury poisoning from working at a mercury thermometer factory in 1988, increased public awareness on this matter. Around this time, multiple cases of mercury poisoning occurred in workers of fluorescent lamp, mercury thermometer, and mercury battery factories [5]. However, no cases of mercury poisoning were reported for the following 10 years. In 2000, the Occupational Safety and Health Research Institute (OSHRI) confirmed mercury poisoning in three workers of an industrial waste recycling company, involved in the process of silver recovery from sludge of semiconductor product [6, 7]. There were no reported cases of occupational mercury poisoning for the following 15 years. However, in March 2015, mercury poisoning occurred in workers who were dismantling a fluorescent lamp factory that had shut down about a month. This incident entered the public domain when two workers filed for industrial accident compensation. At the time, the Ministry of Labor ordered a health examination of the workers. The results showed that 18 out of 21 workers involved in the demolition project had developed a range of symptoms of mercury poisoning up to 6 months after the initial exposure. Now, we report the summary results of our 1-year follow-up of 10 patients with mercury poisoning and two cases with unusual and previously undocumented symptoms.

## Case presentation

Out of a total of 20 cases of mercury poisoning, we present two cases with more severe and unusual symptoms, and show summary results of another cases.

### The summary results of cases

18 out of the 21 workers involved in the demolition project had developed a range of symptoms of mercury poisoning up to 6 months after the initial exposure. After excluding 1 worker who was asymptomatic, we examined 20 workers and followed-up 10 workers with persistent late onset symptoms for more than 1 year.

Early symptoms developed in 18 workers with mercury poisoning were skin rash, pruritus, myalgia, sleep disturbance, cough and sputum production, paresthesia of extremities, fatigue, headache, gastrointestinal symptoms (dyspepsia, nausea, vomiting), tooth and gum symptoms (toothache, moving teeth, gum swelling), and urinary symptoms (Table 1). However, they were not aware that their symptoms indicated mercury exposure, and the physicians who provided initial care also misdiagnosed as a common cold or food poisoning. As a result, chelation therapy was not performed at the early stage.

**Table 1:** Early symptoms of mercury exposure (*n* = 20)

Early symptoms	N	(%)
Skin rash	17	(85%)
Pruritus	9	(45%)
Myalgia	8	(40%)
Sleep disturbance	6	(30%)
Cough or Sputum production	5	(25%)
Paresthesia of the extremities	4	(20%)
Fatigue	4	(20%)
Other skin symptoms (scale, scalding, irritation)	2	(10%)
Headache	2	(10%)
Gastrointestinal symptoms (dyspepsia, nausea, vomiting)	2	(10%)
Tooth and gum symptoms (toothache, moving teeth, gum swelling)	2	(10%)
Urinary symptoms (frequency, incontinence, sensation of incomplete urine voiding)	1	(5%)
Eye symptoms (red eye, decreased visual acuity)	1	(5%)
High blood pressure	1	(5%)

They were exposed to significant amounts of mercury vapor in the underground space. The company requesting the demolition service did not perform any preliminary evaluation of work site or take measurements for mercury exposure, so there were no quantitative reference data to assess the level of mercury exposure at the time of the event. The fluorescent lamp factory was located underground with poor ventilation. According to the workers, the residual pool of mercury within the pipes moved into the underground space during demolition. In the early stages, the smog and the stinging smell in the closed underground space made demolition difficult. Jobs were categorized into management and supervision, cutting, transportation of debris, and driving excavators or lift cranes, and each worker worked for different periods of time. Approximately 200–300 days after completion of the project, the mean urinary mercury concentration was the highest among the cutters, followed by the debris transporters, and the mean urinary mercury concentration among drivers of excavators or high place operating cars was lowest (Table 2).

**Table 2:** Mean and standard deviation of blood mercury and adjusted urinary mercury levels by type of work

Type of work	N	Working day (day(s))		Blood Mercury^a^ (μg/L)		Adjusted Urinary mercury^a^ (μg/L·creatinine)	
		mean (range)		mean (SD)		mean (SD)	
Total	20	10.2	(1–30)	5.88	(4.49)	13.48	(15.40)
Ordering	3	30	(30)	5.67	(3.48)	12.21	(12.84)
Cutting	6	8.5	(1–15)	7.34	(7.87)	22.04	(11.92)
Machinery operating	5	2.4	(1–6)	5.25	(2.27)	0.99	(0.55)
Transporting	6	8.5	(2–15)	5.04	(1.39)	15.95	(21.02)

^a^ Earliest laboratory values preformed within 200–300 days from the last work day

Ordering: instructing somebody who does other type of task to do their task and cleaning work site

Cutting: dismembering facilities and tearing down

Machinery operating: operating machinery vehicle (including forklifts and high place operating cars)

Transporting: manual laborers who transport dismantled facilities and waste

One year following initial exposure to metal mercury vapor, 10 workers exhibited common symptoms of easy fatigue, insomnia, bad dreams, and anxiety disorder. Seven workers are currently undergoing psychiatric treatment for sleep disturbance, anxiety disorder, and depression, and three workers underwent dermatology treatment for hyperpigmentation, erythematous skin eruption, and chloracne-like skin lesions. In particular, worker B developed hyperpigmentation, in addition to chloracne-like lesions, such as hyperkeratosis, epidermal cysts, acne-like eruption of comedones, and granulomatous inflammation involving the face, neck, back, and chest. A further two workers (I and K) developed hyperpigmentation and mercury exanthema, which are skin symptoms. Three workers developed shock-like coarse jerky movements and 2 workers had a swan neck deformity of the finger (workers A and G). Two workers underwent treatment at a pain clinic for paresthesia in the lower limbs, including pain and excessive warm and cold sensations. Two workers were treated at the urology clinic for dysfunction of urologic system with impotence (Table 3).

**Table 3:** Symptoms of 10 patients with late symptoms (>1 year of exposure)

Patient	Age	Task	Working day	Symptoms
A	43	Ordering	30	G: severe fatigue, anorexia, non-specific exertional dyspnea M: mercurial tremor, coarse jerky movement of the right arm and leg, mild swan neck deformity of the right hand 3rd, 4th, and 5th fingers P: anxiety, depression, sleeplessness, bad dreams O: decreased visual acuity, blurred vision, - Consultation: neuropsychiatry & ophthalmology
B (Case 1)	40	Ordering	30	G: severe fatigue, dizziness, non-specific exertional dyspnea M: lower limb swelling D: dark skin patches, epidermal cysts, chloracne like skin lesions, pruritus P: anxiety, depression, sleeplessness, bad dreams, - Consultation: neuropsychiatry and dermatology
E	53	Cutting	7	P: sleeplessness, bad dream, loss of memory
F	52	Cutting	7	G: general myalgia M: thumb muscle weakness and numbness, coarse jerky movement of the lower limb P: sleeplessness, sleep-talking
G (Case 2)	60	Cutting	15	G: easy fatigue M: bilateral pain and paresthesia of the feet, swan neck deformity of both fingers without joint pain, coarse jerky movement of the lower limb P: anxiety, sleeplessness, bad dream U: residual urine sense, urine leak, weak urine stream, impotence - Consultation: neuropsychiatry, urology and pain clinic
I	47	Transporting	8	G: easy fatigue D: hyperpigmentation, urticarial dermatitis in whole body skin P: sleeplessness, bad dream, anxiety, depression, suicidal tendency - Consultation: neuropsychiatry and dermatology
J	50	Transporting	9	P: loss of memory, sleeplessness, headache, anxiety - Consultation: neuropsychiatry
K	46	Transporting	10	D: multiple various sized erythematous patches with itching sense O: decreased visual acuity, blurred vision P: headache, anxiety - Consultation: neuropsychiatry & dermatology
P	55	Cutting	15	G: general myalgia and weakness, non-specific exertional dyspnea, easy fatigue, dizziness M: coarse jerky movement of lower limb, bilateral pain and paresthesia of the feet D: skin rash and itching sense, sleeplessness - Consultation: neuropsychiatry and neurology
S	44	Cutting	6	G: general myalgia, sleeplessness, easy fatigue, dyspepsia, dizziness, gum pain M: edema of the dorsal aspect of the foot, hand, eyelid and neck P: anxiety, sleeplessness, bad dreams U: sensation of incomplete voiding after micturition, urine leak, weak urine flow, impotence - Consultation: urology

*G* general symptoms, *M* neuro-muscular symptoms, *P* central nervous system symptoms, *O* visual symptoms, *U* urologic symptoms

### Worker B

#### Patient information

40-year-old man.

#### Chief complaint

At the time of visiting our Occupational and Environmental Medicine (OEM) clinic (6 months after exposure), worker B’s main symptoms were sleep disturbance and severe fatigue. And he also complained skin rash without itching, as well as mild cough and sputum production.

#### Past history

No remarkable past medical history.

#### Smoking history

Current-smoker, 10 years, 1 pack of cigarettes per day.

#### Alcohol history

3 times per week, 1 bottle of soju. The patient reports drinking owing to sleeping difficulties.

#### Occupational history

The patient had previously worked in the sales department of a manufacturing company and on demolition projects of various facilities for the last 6 years. He was a field manager for the demolition of a fluorescent lamp factory for 30 days from March 15, 2015.

#### Present illness

In October 2015, He became aware of a media report that one of his co-workers involved in the fluorescent lamp factory demolition had been diagnosed with acute mercury poisoning. Therefore, he attended our OEM clinic and was diagnosed with mercury poisoning. He stated that he had developed a general skin rash associated with itching approximately 1 week after commencement of the demolition work. He also developed a mild cough and sputum. However, these symptoms disappeared approximately 3–4 days after completion of the demolition project. He visited our clinic 6 months after the initial mercury exposure complaining of severe insomnia and easy fatigability. Also, He felt difficulty in breathing, even after small movements, and sensation of being drunk, with tingling of the hands, and eye twitching. He reported an intermittent skin rash, accompanied by severe itching of the hands and arms, visual deterioration, anxiety, annoyance, and loss of libido and appetite.

#### Physical examination

Normal sensory and motor functions of the extremities.

#### Laboratory findings

Laboratory test results were as follows: blood mercury level of 15.61 μg/L, urinary mercury level of 4.47 μg/L, and urinary creatinine level of 61.2 mg/dL. Protein was not detected in the urine test. The patient had no sign of kidney damage, with a β2-microglobulin of 1.8 mg/L, blood urea nitrogen (BUN) of 9.80 mg/dL, blood creatinine level of 1.02 mg/dL. Levels of hepatic enzymes were mildly elevated: aspartate aminotransferase (AST) 59.6 U/L, alanine aminotransferase (ALT) 50.9 U/L, γ-glutamic transpeptidase (γ-GTP) 208 U/L. Chest radiographs were normal. Pulmonary function tests were performed, although we failed to obtain reliable and valid results owing to breathing difficulty.

#### Progression

**Fig. 1:**
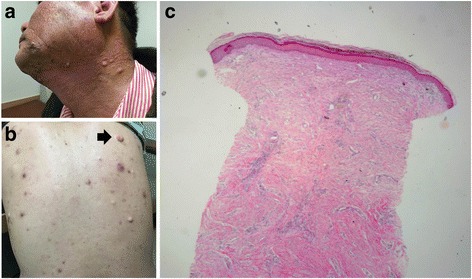
Skin lesions and histopathology of worker B. **a**. hyperpigmentation, multiple cysts, chloracne-like lesions of face and neck,** b**. soft cysts with yellowish pus and papules with comedones (*arrow*: biopsy site), **c**. Histopathologic findings showed chronic inflammation with dense fibrosis, chronic granulomatous inflammation, hyperkeratosis (hematoxylin and eosin stain, × 40)

Nine months following mercury exposure, the patient’s face was hyperpigmented and multiple cysts developed on the back and neck area. He lost approximately 10 kg of body weight (from 102 kg to 92 kg). He reported a persisting sharp pain and tingling sensation in both arms. He complained of frequent bad dreams and became anxious. The patient reported extreme anxiety with a feeling of being suffocated when riding the elevator alone or inside a theater. We prescribed antidepressants to control these symptoms, treatment was ineffective. The patient was referred to the mental health clinic for drug and psychological therapy. Six months after attending our clinic for the first time (12 months after exposure), he was referred to the dermatology clinic for multiple lumps and cysts on the face and upper back. Biopsies were taken for a suspected chloracne and epidermal cyst. The findings indicated epidermal cysts, hyperkeratosis, increased basal pigmentation, chronic perivascular inflammation, chronic granulomatous inflammation, and pigmented macrophages in dermis (Figure 1). Injection therapy was performed to treat the keloid scar that developed in the chest area, and both punch biopsy and squeezing were performed to treat the cysts. Currently, symptomatic drug treatment is administered for the chloracne, but symptoms continue to fluctuate between exacerbations and improvement.

### Worker G

#### Patient information

60-year-old man.

#### Chief complaint

The patient developed a skin rash without itching, headache, toothache, and myalgia in the early stages of mercury exposure. Numbness and pain of the right leg worsened after 1–2 weeks of completion of the demolition. After that, he complained of sleep disturbance, anxiety, sense of incomplete voiding after micturition, and erectile dysfunction.

#### Past history

The patient had a history of hypertension, but no history of diabetes, hepatitis, thyroid diseases, or pulmonary pneumonia.

#### Smoking history

Current-smoker, 35-year history of smoking, 1.2 pack of cigarettes per day.

#### Alcohol history

3 times per week, 1 bottle of soju (quit drinking liquor after symptoms started).

#### Occupational history

The patient had previously worked in the delivery department of an ampule and vial manufacturer. He had worked in the demolition industry for the last 10 years. For 2 weeks, starting on March 23 2015, he performed oxygen cutting in the fluorescent lamp factory.

#### Present illness

The patient developed a general rash, myalgia, headache, and toothache while working on the demolition project and attributed the symptoms to the common cold. After completing the demolition project in mid-April, he developed tingling in the right foot and formication, and an exacerbation of his headache and toothache. The skin rash repeatedly appeared and disappeared. Following a diagnosis of potential heavy metal poisoning, made at a clinic of oriental medicine, the patient visited a university hospital for heavy metal testing. The results confirmed high levels of mercury, with a blood mercury levels of 16.386 μg/L and a urinary mercury levels of 161.775 μg/L, 2 months following cessation of exposure to mercury. Despite symptomatic treatment, including vitamins, non-steroidal anti-inflammatory drugs (NSAIDs), neuropathic pain relievers, and nerve block, the insomnia and tremor deteriorated, and the patient developed hypesthesia and palsy in the hands. Proteinuria was confirmed by a urine test. Five months after mercury exposure, the patient was transferred to a large general hospital in Seoul to undergo digital infrared thermal imaging (DITI) and a bone scan. Both tests showed no abnormal findings, but the pain persisted. He underwent a nerve block at a pain clinic, but the symptoms worsened, progressing to weakness of muscles, pain, and paresthesia in the lower limbs that hindered ambulation.

Seven months after exposure to mercury, the patient was transferred to our clinic. Major symptoms were insomnia and pain, paresthesia (tingling/aching and flushing), and edema of the lower limbs. The patient also reported a sudden electric shock-like sensation in the both right foot and right hand, described as like being hit by lightning. This generally lasted for approximately 1 min. Owing to pain, the patient reported difficulty walking for more than 200 m in distance, dyspnea, paresthesia (described as the sensation of a bug crawling over the skin), backache, and toothache. Despite symptomatic treatment, these symptoms repeatedly fluctuated between improvement and exacerbation, according to his general condition.

#### Physical examination

**Fig. 2:**
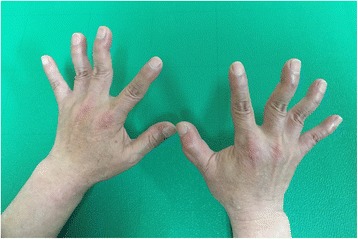
Swan-neck deformity of both fingers, worker G

The patient also reported muscle weakness of the upper and lower limbs. And a bilateral swan-neck deformity developed on 3rd and 4th fingers (Figure 2). Furthermore, shortening, tightness, and decreased grip power of both finger extensors were observed.

#### Laboratory findings

Laboratory findings at 7 months following cessation of mercury exposure were as follows: blood mercury level of 4.26 μg/L, urinary mercury level of 39.22 μg/L, urinary creatinine level of 126 mg/dL, and proteinuria 2+. Signs of kidney injury included: a β2-microglobulin level of 1.8 mg/L, N-acetyl-β-glucosamidase level of 5.45 IU/g·creatinine (NAG index: 0.02–3.65 U/g in male), protein level (urine) of 22.7 mg/L, protein-to-creatinine ratio in spot urine sample of 0.21, BUN of 9.80 mg/dL, blood creatinine level of 1.02 mg/dL. Hepatic enzymes were also elevated: AST 59.6 U/L, ALT 50.9 U/L, γ-GTP 208 U/L.

#### Progression

Ten months after the end of mercury exposure, drug therapy led to improvements in pain and paresthesia in the lower limbs, although symptoms were exacerbated following interruption of the drug therapy. Despite sleep medication, the patient only achieved 3–4 h of sleep per night. Duloxetine was prescribed because the patient complained of anxiety. His renal ultrasound showed a “small right kidney with decreased size of the parenchyma.” Proteinuria persisted and an angiotensin II receptor blocker (ARB) was prescribed.

Anxiety disorder and sleep disturbance persisted for 13 months after exposure, so the patient concomitantly underwent drug and psychological therapy at a mental health clinic. Proteinuria improved, although the patient reported a sense of incomplete voiding after micturition, urine leak, poor urine flow, and impotence. Urodynamic studies performed at the urology clinic indicated dysfunction with no structural abnormality. Sildenafil was administered for the treatment of impotence, which led to erection but no ejaculation. The patient’s body weight decreased to 45 kg owing to loss of appetite. This subsequently increased to 55 kg, 3 kg below his body weight before the mercury poisoning incident.

## Discussion and conclusions

### Early symptoms of mercury poisoning

Exposure to metal mercury vapor generally affects the respiratory system, digestive system, kidney, skin and mucus, and nervous system [8, 9]. Fatal chemical pneumonitis can occur under the high concentration (>1 mg/m3) exposure of mercury vapor [10]. In the early stages of exposure to mercury vapor, patients may report a metallic taste in the mouth or develop stomatitis. Digestive symptoms include nausea, vomiting, abdominal pain, and diarrhea. The kidney is a major target organ, and mercury exposure can induce proteinuria, oliguria, and nephritis. Hepatitis may lead to elevated levels of hepatic enzymes. Skin symptoms include general erythematous skin eruptions, with itching and hyperhidrosis. In children, acrodynia may rarely occur, which is characterized by desquamation and erythema in the palms and soles of feet [11]. Typical, previously reported symptoms developed in 18 workers with mercury poisoning (Table 1).

### Late symptoms of mercury poisoning

The nervous system suffers the greatest damage from the accumulation of metal mercury within the body. Erethism, characterized by increased irritability, lack of patience, avoidance of people, excessive shyness, and insomnia, is a well-established symptom of metal mercury poisoning [12]. Mercury poisoning may also induce flapping tremor, initially occurring as a resting tremor that progresses to intention tremor, accompanied by a rough and rhythmic movement. At first, the tremor usually involves the hands and later it affects the eyelids, lips, tongue, and head. Furthermore, cognitive impairment, such as dystaxia, and disturbances in memory and attention, may develop [13, 14]. Frequent episodes of coarse jerky movements are also typical symptoms associated with mercury poisoning [14]. Mercury is a well-known nephrotoxic substance. Its toxicity ranges from mild changes in urine acidity, mild albuminuria, and proteinuria to nephrotic syndrome and renal failure, accompanied by proximal tubular necrosis [13, 15, 16]. Multiple skin diseases have also been associated with mercury exposure. Acrodynia, characterized by flare-up, pain, and peeling of skin in the acral regions (e.g., tips of hands and feet), usually develops in children after prolonged mercury exposure. Furthermore, contact dermatitis and hyperpigmentation have also been commonly reported after exposure to mercury-containing substances [14, 17]. Despite of a debate whether the skin symptoms of worker B is due to mercury, there are some probabilities of mercury-related symptom considering that the skin hyperpigmentation, which is known for post-mercury-exposure symptom, was occurred and skin immunity could be affected by mercury. Swan neck deformity is generally a manifestation of rheumatoid arthritis or a posttraumatic sequela [18]. However, worker G did not have these conditions nor did he specifically complain of pain in the joints. The authors assume this condition as the motor-sensory incoordination of the hand.

### Level of exposure to mercury vapor

The nervous system is generally affected by exposure to air mercury concentrations greater than 0.1 mg/m^3^ [19]; however, there is evidence of nervous system symptoms in workers with chronic exposure to concentrations of mercury vapor which are 2–4 times lower [20, 21]. The following renal symptoms have been documented at air mercury concentrations of 0.02–0.45 mg/m^3^: proteinuria, proximal tubular necrosis, and sclerotic changes of the glomerulus [22]. Furthermore, it has been reported that respiratory symptoms may develop after hours of exposure to relatively higher air mercury concentrations, around 1–3 mg/m^3^ [10, 23]. The majority of patients presented here manifested nervous system symptoms with mild respiratory symptoms. Therefore, we can estimate a level of mercury exposure of 0.1–1 mg/m^3^.

### Reason for lack of improvements of symptoms

One year after the initial exposure to mercury, symptoms were present in 10 out of the 18 workers with early symptoms. This may be owing to mercury being captured by the central nervous system. Metal mercury vapor enters the blood stream after absorption through the respiratory tract. It then penetrates the blood-brain barrier, accumulating in brain tissues. When elemental mercury in the brain tissues is oxidized, it cannot cross the blood-brain barrier. Therefore, it remains in the brain tissues for prolonged periods [24, 25]. It has been reported that the half-life of accumulated mercury may range from several years to several decades [26]. Furthermore, chelation therapy using dimercaptosuccinic acid (DMSA) and mercaptopropane sulfonate (DMPS) hardly reduces mercury concentrations in brain tissues [27]. We unsuccessfully attempted chelation therapy in 4 patients; therapy was discontinued owing to side effects, such as nausea. In conclusion, early chelation therapy could not be performed in these cases owing to the delayed diagnosis of mercury poisoning. Considering that patients did not show marked improvement of symptoms over the last year, we predict that their symptoms will become chronic.

## Abbreviations

ALT: Alanine aminotransferase
ARB: Angiotensin all receptor blocker
ASGM: Artisanal and Small-scale Gold Mining
AST: Aspartate aminotransferase
BUN: Blood urea nitrogen
CFL: Compact fluorescent lamp
c-GTP: γ-glutamic transpeptidase
DITI: Digital infrared thermal imaging
DMPS: Mercaptopropane sulfonate
DMSA: Dimercaptosuccinic acid
LED: Light emitting diode
NSAIDs: Non-steroidal anti-inflammatory drugs
OEM: Occupational and Environmental Medicine
OSHRI: Occupational Safety and Health Research Institute
UNEP: United Nations Environment Programme.
